# Ophthalmic and Visual System Changes in Human Spaceflight: A Review of Mechanisms, Measurement, and Countermeasures

**DOI:** 10.3390/jcm15124537

**Published:** 2026-06-11

**Authors:** Natalia Lange, Filip Wylęgała, Bartłomiej Bolek, Bogumiła Sędziak-Marcinek, Jarosław Piłat, Edward Wylęgała, Adam Wylęgała

**Affiliations:** 1 Chair and Clinical Department of Ophthalmology, School of Medicine in Zabrze, District Railway Hospital, Panewnicka 65, 40-760 Katowice, Poland; 2 Faculty of Space Technologies, AGH University of Krakow, Mickiewicza 30, 30-059 Krakow, Poland; 3 Experimental Ophthalmology Unit, Department of Biophysics, Medical University of Silesia, Panewnicka 65, 40-760 Katowice, Poland

**Keywords:** spaceflight, microgravity, SANS, ocular complications, optical coherence tomography, radiation, cataract

## Abstract

**Background**: Long-duration spaceflight (LDSF) poses unique challenges to ocular health as microgravity, radiation, and environmental changes can cause lasting visual and structural impairments that affect astronaut performance. **Objective**: This review synthesises current evidence on in- and post-flight ocular complications. It integrates clinical findings, terrestrial analogues, animal studies, and theoretical models to characterise the pathophysiology, risk factors, and countermeasures associated with spaceflight-induced ocular changes. **Methods**: A review of peer-reviewed literature was conducted, focusing on dry eye disease, corneal edema, ocular biometric shifts, spaceflight associated neuro-ocular syndrome (SANS), and radiation-induced cataractogenesis. Data from in-flight imaging, post-flight assessments, and ground-based analogues were analysed. **Results**: Spaceflight induces multifactorial ocular changes, including tear film instability, optic disc edema, posterior globe flattening, and hyperopic refractive shifts. These effects are thought to result from cephalad fluid shifts compartmentalised cerebrospinal fluid pressure, venous congestion, and impaired glymphatic system. Long-term risks, such as cataractogenesis, are linked to radiation exposure and genetic susceptibility. Although several countermeasures are being explored, no single approach fully prevents these complications. **Conclusions**: Ocular complications during LDSF remain a significant challenge for astronaut health and mission performance. A multimodal approach combining mechanical, nutritional, and diagnostic strategies will be essential for future exploration-class missions. Further research is needed to refine countermeasures and preserve astronauts’ visual function.

## 1. Introduction

Spaceflight presents a uniquely hostile environment for human physiology. Microgravity, radiation, and altered atmospheric conditions profoundly affect multiple organ systems. Among these, the ocular system has emerged as particularly vulnerable with astronauts experiencing both acute and chronic visual changes. Disorders ranging from dry eye disease and biometric shifts to optic disc swelling can threaten performance and long-term ocular health [1,2,3,4,5].

As exploration-class missions to the Moon and Mars become increasingly feasible, understanding the mechanisms, risk factors, and clinical manifestations of spaceflight-induced ocular complications is essential. This review synthesises current knowledge from terrestrial analogues, in-flight observations, and post-flight assessments [6,7] to provide a comprehensive overview of ocular changes. By integrating imaging [8,9,10], molecular [11,12,13,14,15,16], and biomechanical data [17,18], we aim to clarify the pathophysiology of these conditions and evaluate the efficacy of emerging countermeasures [4,19,20]. Ultimately, this work seeks to inform future medical protocols and safeguard astronaut vision in the era of deep-space exploration.

## 2. Methods

This review synthesizes peer-reviewed evidence on in-flight and post-flight ocular complications associated with long-duration spaceflight and evaluates their implications for spacecraft/habitat design, in-flight monitoring, and countermeasure development. A literature search was conducted using PubMed/MEDLINE, Scopus, and Web of Science databases. English-language articles published between January 2006 and December 2025 were screened for relevance using predefined inclusion criteria. Study selection followed PRISMA guidelines. Full search strategies and PRISMA details are provided in Appendix A.

## 3. Spaceflight Drivers and Design Variables

### 3.1. Microgravity Physiology and Fluid Shifts

Microgravity eliminates the Earth-derived hydrostatic pressure gradient (≈70 mmHg at the head vs ≈200 mmHg in the lower limbs) [21]. As a result, blood and interstitial fluid shift cephalad, redistributing arterial pressure toward the upper body [21]. This shift is sensed by cervical baroreceptors, which trigger reflex vasodilation and reductions in heart rate and mean arterial pressure [21]. This fluid-dynamic change is considered to be a primary driver of posterior-segment remodeling and the ophthalmic manifestations of spaceflight associated neuro-ocular syndrome (SANS) [5,22].

**Figure 1:**
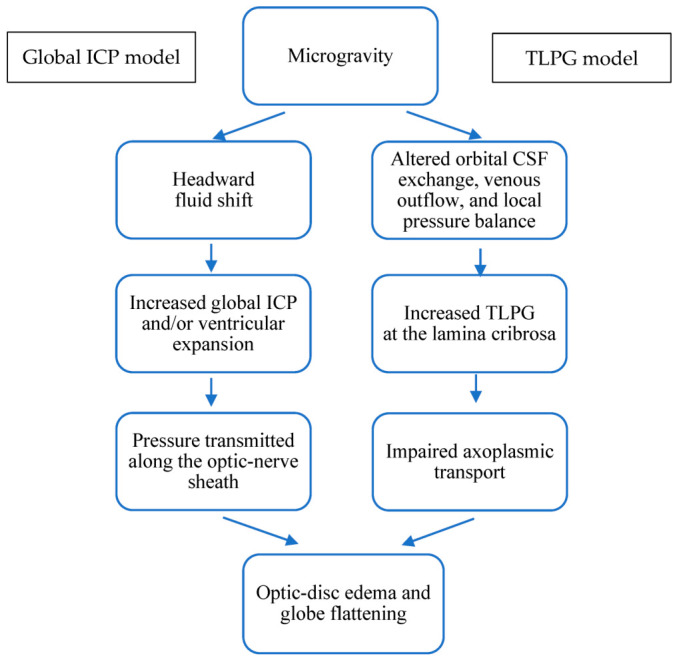
Proposed ICP- and TLPG-mediated pathways underlying Spaceflight Associated Neuro-ocular Syndrome (SANS) during microgravity-induced fluid shifts.

The classic intracranial-pressure (ICP) hypothesis posits that head-ward fluid shifts raise ICP and expand ventricular volume (Figure 1) [23,24,25]. Excess pressure may then travel along the optic-nerve sheath, causing axoplasmic stasis, globe flattening, and optic-disc edema [19]. Experimental elevation of ICP to 30 mmHg in murine models produces retinal-ganglion-cell loss and optic-nerve disorganization, supporting a causal link [26]. However, clinical observations challenge a simple ICP-driven model, and several studies suggested that global ventricular expansion is not a mandatory indicator of the syndrome. Post-flight lumbar-puncture data often show only borderline ICP [8,22]. SANS findings, such as unilateral edema, choroidal folds, and persistent ocular deformation even after opening pressure normalizes, diverge from classic idiopathic intracranial hypertension and challenge an ICP only explanation [8,22]. For instance, opening pressures have been observed to drop from a borderline-elevated 22 cm H_2_O shortly after mission completion to a baseline of 16 cm H_2_O one year later, while ocular deformations remained present [27]. Additionally, neuroimaging shows that astronauts with SANS may exhibit smaller ventricular expansions than unaffected crew, suggesting compartmentalized rather than global fluid shifts [28].

An alternative SANS hypothesis emphasizes the translaminar pressure gradient (TLPG) rather than ICP alone: altered cerebrospinal fluid (CSF), venous sinus pressure and cerebral blood flow, may disrupt axonal transport at the optic nerve head (ONH), making this gradient a primary determinant of axonal health [29,30,31]. Therefore, optic disc edema in SANS may represent a mechanical consequence of altered pressure balance across the lamina cribrosa, leading to disruption of axoplasmic transport (Figure 1).

In short, the global ICP model treats SANS as a brain-wide pressure problem: if overall cranial CSF pressure rises sufficiently, ocular changes follow. The compartmentalized CSF/TLPG model instead treats SANS as a local pressure-balance problem at the ONH, where abnormal CSF clearance, venous congestion, or pressure gradients within the optic-nerve sheath could produce persistent eye findings despite only modest or transient ICP elevation.

Moreover, the loss of gravity abolishes the “pumping” action generated by eye movements that ordinarily facilitates cerebrospinal-fluid exchange between the orbital subarachnoid space and the intracranial compartment, further impairing clearance pathways [32].

### 3.2. Intraocular Pressure Dynamics Under Microgravity: Acute Responses, Adaptation, and Clinical Implications

Although intraocular pressure (IOP) is not part of the formal definition of space-flight-associated neuro-ocular syndrome (SANS), it is a sensitive indicator of ocular fluid dynamics and its interaction with intracranial pressure [25,33].

Upon entry into microgravity, IOP rises within minutes [33,34]—a similar immediate increase is observed after as little as 15 min of head-down-tilt (HDT) bed rest [35]. This suggests that the loss of the hydrostatic gradient quickly elevates episcleral venous pressure, transiently pushing the aqueous-humor column forward [35,36]. IOP usually returns to baseline within a week, and some astronauts even exhibit a modest post-flight decrease [33,34]. Notably, individual variability exists: the first Korean astronaut showed a sustained 26% IOP rise through mission day 8 and a reversal of the normal diurnal rhythm [36]. Animal models mirror these transient adaptations. Hindlimb-unloaded mice exhibit a distinct ocular phenotype in which IOP peaks at day 14 before undergoing an adaptive decline, closely paralleling early-mission IOP fluctuations reported in astronauts [37].

Complementary head-down tilt experiments demonstrate that intraocular and episcleral venous pressures increase modestly and in parallel, despite substantially larger cephalic venous pressure shifts and unchanged aqueous humor production [35,38]. Those results indicate the presence of intrinsic ocular buffering mechanisms that limit excessive IOP elevation under microgravity-like conditions [35,38]. Most documented SANS cases occur with IOP values within the normal range, and current evidence does not identify sustained IOP elevation as an independent risk factor. Instead, the critical variable appears to be the TLPG between IOP and CSF pressure at the lamina cribrosa as presented in Equation [31]. Translaminar pressure gradient (TLPG) is directly proportional to the difference between intraocular pressure (IOP) and cerebrospinal fluid pressure (CSFP), and inversely proportional to the lamina cribrosa thickness (LC) (1) [31,39].

(1) $$TLPG=\frac{IOP-CSFP}{LC}$$

In this context, countermeasure concepts that deliberately induce modest IOP elevations, such as exercise or swimming goggles, have been explored to counterbalance increased optic nerve sheath or CSF pressure, thereby reducing the TLPG and potentially mitigating optic disc edema [40].

To quantify cephalad fluid redistribution, the Thoracic Fluid Index (TFI) has been employed. It is a cardiovascular metric of cephalad fluid shift, which varies in direct proportion to IOP elevation in microgravity simulations, linking systemic fluid redistribution to ocular pressure changes [41]. The proportional relationship between TFI and IOP under simulated microgravity makes TFI a practical, non-invasive marker for cephalad fluid shifts that could be tracked continuously to flag a widening TLPG, especially when IOP appears deceptively stable. For astronauts with known glaucoma risk factors or ocular hypertension, the acute IOP rise upon entering microgravity may pose additional hazard, though no cases of glaucomatous progression have been documented in-flight.

Taken together, these data support the view that IOP behaves as an early, largely reversible marker of microgravity-induced fluid redistribution and episcleral venous pressure, rather than a primary driver of SANS [33].

## 4. Anterior Segment Complications in Spaceflight: Risk Factors and Mechanisms

### 4.1. Types of Anterior Segment Complications

In a retrospective case series ocular events were reported in 83 of 135 Space Shuttle (STS) missions from the years 1981 to 2011, as well as 41 out of 63 ISS missions from the year 2000 to 2020 [42,43]. A total of 242 complaints were documented, most commonly eye irritation (33.1%), foreign body sensation (22.7%), dry eye (15.7%), and epiphora (7.9%). Severe cases, including keratitis, corneal ulcer, and abrasion, comprised 3.7% of complaints. 5.2% of them occurred during or immediately after extravehicular activities (EVAs), largely due to eye irritation from particulates or antifog agents, all of which resolved after helmet removal or symptomatic treatment [43].

#### 4.1.1. Spaceflight Associated Dry Eye Syndrome (SADES)

Dry eye syndrome (DES) is among the most frequent ocular surface complaints during space travel, affecting approximately 20–30% of astronauts [42,44], and it has the potential to impact both astronaut well-being and operational performance. In spaceflight associated dry eye strong cabin airflow, elevated CO_2_, and radiation accelerate tear evaporation. At the same time, microgravity-induced periorbital edema, altered eyelid position and blink efficiency, meibomian gland dysfunction, and immune dysregulation collectively destabilize the tear film and weaken ocular surface defenses, leading to a predominantly evaporative and inflammatory dry eye state as shown in Table 1 [1,45,46]. Astronauts describe a spectrum of symptoms, ranging from eye irritation and the sensation of a foreign body to keratoconjunctivitis sicca, periorbital edema, and excessive tearing [43]. These symptoms can compromise comfort, visual function, and ultimately performance during critical tasks.

#### 4.1.2. Corneal Edema

Corneal edema, although not yet documented in astronauts in vivo [47], represents a plausible risk to the anterior segment during spaceflight, given the confluence of environmental and physiological challenges. A retrospective study of central corneal thickness (CCT) after spaceflight, including astronauts who had undergone photorefractive keratectomy or LASIK, found no significant differences between pre- and post-flight measurements [47,48,49,50]. On Earth and in ex vivo settings, the reported risk factors include contact lens-related hypoxia, corneal conditions or disorders, and molecular insults [47].

In terrestrial ophthalmology, marked acute elevations in IOP can cause corneal stromal edema by overwhelming endothelial pump function [51]. However, such extreme or sustained IOP elevations have not been documented during spaceflight, so this mechanism remains theoretical for astronauts. More plausible drivers of stromal swelling in the spaceflight context are oxidative and IL-8-mediated stress, as well as cytoskeletal dysfunction [47,52].

While ultrasound pachymetry remains the gold standard for measuring CCT, spectral domain anterior-segment optical coherence tomography (AS-OCT) is emerging as a more practical diagnostic tool aboard the ISS due to its integration into existing imaging platforms [47].

### 4.2. Risk Factors

Due to environmental conditions that differ from those on Earth, spaceflight may increase the risk of anterior segment complications through various mechanisms.

#### 4.2.1. Cabin Environment and Tear-Film Destabilization

Spacecraft environment and the physiological effects of microgravity converge to significantly destabilize the tear film, compromising ocular surface health. Atmospheric factors, including high air exchange rates of 45–77 times per hour, together with a relative humidity of about 60% and consistently elevated levels of CO_2_, create conditions that accelerate tear evaporation [1,53,54], predisposing astronauts to dry eye syndrome.

#### 4.2.2. Microgravity-Induced Oculofacial and Visual Changes

Physically, microgravity-induced fluid shifts and the loss of gravitational drag result in periorbital edema, ‘reverse ptosis’ of the lower eyelid, and a coordinated 2.3–2.4 mm elevation of the eyebrow, which can distort the tear meniscus and obstruct the visual field [55]. Long-duration missions further exacerbate these issues by weakening the orbicularis oculi muscle, which impairs blinking efficiency [56] and disrupts the secretion of essential lipids from the meibomian glands [45]. The consistent elevation of the brow and eyelid suggests a predictable, unified neuromuscular adaptation to the absence of gravity, necessitating a shift in how visual field obstructions are managed in orbit [55].

Beyond the cabin environment, microgravity alters eye movement and visual focus, disrupting the vestibular system and dynamic visual acuity [23,57]. Without reliable gravitational cues, otolith signaling is altered and the brain compensates by down-weighting vestibular input in favour of visual and somatosensory feedback [58,59].

In parallel, astronauts participating in EVAs face the risk of ocular decompression sickness, a form of decompression illness that occurs when exposure to a hyperbaric or hypobaric environment causes inert gases, most commonly nitrogen, to come out of solution and form bubbles within the ocular vasculature and surrounding tissues [60]. It may be mitigated through intensive pre-breathing protocols and targeted aerobic exercises to purge nitrogen from the vascular and interstitial ocular compartments [61].

#### 4.2.3. Radiation Environment Across Mission Profiles

Ionising radiation is a long-term risk factor for ocular pathology. Mission profile and spacecraft shielding strongly influence cumulative exposure, particularly during exploration-class missions beyond low Earth orbit. Beyond permanent structural risks such as caractogenesis, exposure to radiation, mainly galactic cosmic rays and solar particle events, introduces further risks for xerophthalmia [11,21,46]. The long-term, lens-specific consequences of space radiation exposure, particularly cataractogenesis, are discussed in detail in the subsequent cataract section.

Simultaneously, over 80% of astronauts report phosphenes, transient light flashes triggered by a dual mechanism of direct retinal interaction with heavy nuclei and proton-induced knock-on particles [62]. These positive visual phenomena frequently manifest during sleep preparation and may pose an operational safety risk by causing abnormal activation of the visual pathways during critical mission phases [62].

#### 4.2.4. Extraterrestrial Dust, Foreign Body Exposure, and Systemic Factors Increasing Dry Eye Susceptibility

Another serious concern is exposure to extraterrestrial dust. Analysis of mission logs from 1961 to 2020 reveals that nearly 70% of all recorded ocular complaints in space were due to foreign body abrasions, underscoring the constant threat posed by jagged, airborne particulates in the microgravity environment [61]. Lunar dust at concentrations as low as 20 mg/m^3^ was shown to trigger corneal molecular responses [45], while Martian dust, rich in reactive perchlorates, may be even more toxic [1]. However, in vivo and in vitro assessments of authentic Apollo 14 samples indicate that lunar regolith is only minimally irritating, with minor conjunctival inflammation resolving within 24 h and a notable absence of corneal abrasions [63]. These results classify authentic lunar dust as a nuisance particulate rather than a severe chemical hazard. However, micro-abrasions on the skin allow dust to penetrate and irritate the ocular surface [42], and the risk may be elevated for contact lens wearers due to mechanical entrapment [63].

Broader systemic influences compound these local stressors. Effects of spaceflight on the nervous system, such as disruption of the circadian rhythm, sleep and mood disorders, and accelerated aging [1,46,57,64,65], all interact to heighten susceptibility to SADES.

#### 4.2.5. Ocular Infection and Viral Reactivation in the Spaceflight Immune Milieu

Spaceflight produces a pro-infective milieu: microgravity, ionizing radiation, and chronic mission stressors suppress both innate and adaptive immunity, thereby increasing the risk of both new infections and viral reactivation in the crew [66]. Within this context, altered tear-film dynamics in microgravity further compromise ocular defense and clearance, and elevations in systemic cytokines (TNF, IL-1α, IL-1β) have been documented in astronauts, creating conditions that favor the reactivation of herpetic viruses and ocular surface infection [67,68,69]. Beyond general inflammation, the consistent elevation of IL-1ra during missions suggests a specific adaptive response intended to neutralize the pro-inflammatory effects of IL-1 in the microgravity environment [70].

### 4.3. Countermeasures for Anterior-Segment Complications

The current treatment of SADES aboard the ISS primarily relies on artificial tears and lubricants, such as carboxymethylcellulose, hypromellose, and mineral oil ointments, however, these are limited by short retention and logistical challenges in microgravity [2,71]. To address traumatic injuries in-flight, the ISS is equipped with a specialized medical kit containing broad-spectrum antibiotics, such as moxifloxacin, ensuring that corneal ulcers or perforations can be stabilized when immediate return to Earth is not possible [61]. Artificial tears are difficult to administer in microgravity [44] and conventional systemic drugs are limited by altered pharmacokinetics and poor retention on the ocular surface [2].

Emerging countermeasures seek to overcome these limitations but remain investigational and have not yet been validated as operational treatments for SADES during spaceflight. Intranasal neurostimulation leverages the nasolacrimal reflex to increase secretion of aqueous, mucin, and lipid components of the tear film, thereby promoting tear-film homeostasis and offering a reusable, non-expiring modality suitable for long-duration missions [44]. Neurostimulation has been tested during a parabolic flight, reliably producing perceptible tearing in all participants and presenting no safety issues in the microgravity analogue setting [72].

Further SADES countermeasures, including nanomedicine-based formulations, remain largely conceptual or are still in early preclinical development. These formulations, such as liposomes, nanoemulsions, dendrimers, and solid lipid nanoparticles, can improve drug stability, solubility, and ocular surface retention. Chitosan-based systems provide additional mucoadhesive and antimicrobial benefits [2]. Other emerging approaches include, microneedle implants and biosensor-equipped, drug-eluting contact lenses offer controlled, extended delivery tailored to the altered tear dynamics of microgravity, where pooling and impaired drainage are common [67].

Corneal edema countermeasures under consideration would include high-oxygen-transmissible contact lenses, air or gas injections, topical steroids, and hypertonic saline drops [52]. However, given current constraints in surgical infrastructure, microgravity-compatible technology, and access to specialized personnel a definitive treatment with corneal transplantation remain infeasible during spaceflight [52].

Collectively, these approaches underscore that anterior-segment pathology in space, particularly SADES, is a complex, mission-relevant problem, and that the development of robust, scalable countermeasures will be critical for safeguarding astronaut vision and sustaining performance on future lunar and Martian expeditions.

## 5. Microgravity-Induced Ocular Structural Remodeling and Refractive Consequences

Spaceflight alters ocular structure through the combined effects of microgravity, cephalad fluid shifts, and pressure imbalances between the brain and globe. These biomechanical perturbations underlie many of the refractive alterations and structural features of spaceflight associated neuro-ocular syndrome (SANS).

One of the most consistent findings in LDSF is that global ocular geometry is modified: small but measurable reductions in axial length, on the order of 0.05–0.08 mm, have been detected after flight and can persist for up to a year [73], thereby contributing to the hyperopic refractive shifts reported in SANS [74,75]. However, refractive outcomes vary considerably between astronauts. Some eyes become more hyperopic, others show no change, and a minority even develop mild myopia, with baseline myopic eyes being most prone to hyperopic drift [74]. The variability points to a multifactorial process in which age, sex, and genetic variation may all play a role. Terrestrial data show that choroidal and scleral stiffness increase with age [76], meaning older astronauts might exhibit slower vascular engorgement or less structural deformation, though age has not yet been isolated as an independent predictor in the small SANS literature. Female astronauts have historically been underrepresented in SANS studies, and no sex-specific analysis of refractive shift is available.

Another common finding in LDSF is thickening of the tissues around the optic nerve head (ONH). During ISS missions, astronauts exhibit progressive increases in peripapillary choroidal thickness and rim tissue volume, with maximal changes often observed near the end of six-month flights [73]. These alterations are thought to reflect cephalad fluid redistribution and impaired venous outflow, both of which elevate tissue pressure around the optic disc and promote vasodilation [24]. Wåhlin et al. further observed that spaceflight is associated with elongation of the optic nerve (0.80 ± 0.74 mm), driven mainly by forward displacement of the ONH, a change that correlates with mission duration, preflight body weight, and clinical manifestations of SANS [77].

While peripapillary tissues thicken, prospective cohort data indicate that central macular thickness decreases by approximately 5.1 μm, a reversible change that may be driven by compressive forces from chronic IOP elevation or choroidal engorgement [75]. This dissociation between peripapillary thickening and macular thinning suggests that the posterior segment responds to spaceflight in region-specific ways, rather than uniformly.

Short-term terrestrial models offer a useful contrast to the cumulative changes. Acute 40° head-down tilt produces significant but transient choroidal thickening that begins to normalize within ten minutes, consistent with an early vascular autoregulatory response rather than cumulative tissue remodeling [78]. Similarly, murine models employing 30° tail suspension show that cephalad fluid shifts can induce transient retinal microvascular tortuosity and ONH expansion that fully resolve within 30 days through physiological adaptation [79]. This recovery capacity contrasts sharply with the cumulative, and sometimes persistent, structural remodeling observed in long-duration crew members [77].

The effects of microgravity are not confined to the posterior eye. Theoretical models suggest that fluid shifts in the anterior choroid and ciliary body may drive anterior lens displacement and a reduction in anterior chamber volume, contributing further to the hyperopic refractive shifts observed in SANS [80].

These structural changes carry meaningful functional implications as shown in Table 2. Globe flattening, retinal thickening, and ONH swelling may all impair fine visual acuity. Beyond static vision, decrements in dynamic visual acuity, the ability to visualise objects either during head movement or objects in motion, during G-transitions demonstrate that structural changes are accompanied by functional consequences similar to those seen in patients with vestibular disorders [81].

Together, this body of evidence supports a coherent model in which microgravity induces fluid-related remodelling of the posterior eye, resulting in small but measurable biometric changes that accumulate with mission duration and contribute to the clinical spectrum of SANS.

## 6. Spaceflight Associated Neuro-Ocular Syndrome (SANS)—The Clinical Spectrum

The clinical spectrum of spaceflight associated neuro-ocular syndrome (SANS) encompasses a constellation of structural and functional changes that primarily affect near vision [25]. The most frequently reported symptom, as mentioned in the previous paragraph, is progressive hyperopic refractive error shifts of +0.50 D up to +2.75 D, a hallmark finding observed in multiple long-duration missions [4,5,22,83]. Apart from the hyperopic shift, ocular abnormalities that define diagnostic criteria of SANS include unilateral or bilateral optic disc edema (ODE) of variable Frisén grades, chorioretinal folds, and posterior globe flattening [8,22,83]. Although other common abnormalities, including retinal hemorrhages and cotton-wool spots, which indicate localized retinal nerve fibre layer (RNFL) infarction, continue to be monitored, they are no longer considered defining diagnostic criteria [84]. Among chorioretinal folds, choroidal folds were the most common type to develop during LDSF missions to the ISS, affecting 6 out of 36 crewmembers [85].

### 6.1. Time Course and Clinical Progression of SANS

The time course of SANS is closely tied to the duration of microgravity exposure. Symptoms are typically reported during LDSF and may emerge progressively over weeks to months of spaceflight, with some neuro-ocular changes documented as early as ~3 weeks after launch and increased prevalence observed in missions lasting ~4–6 months [83,86,87].

The earliest and most consistent finding is optic disc edema (ODE), which is usually subtle and Grade 1 on the Frisén scale in over 90% of cases. This condition can be detected in-flight after several weeks to months aboard the International Space Station and may persist for up to 180 days [9,34]. Only about 15% of astronauts develop an optic disc edema visible on fundoscopy (Frisén grade ≥1) [88].

Longitudinal OCT data from ISS missions demonstrate that minimal optic disc edema at approximately flight day 30 (ΔTRT < 20 µm) predicts a very low likelihood of clinically significant edema (ΔTRT ≥55 µm) by mid- to late-mission (around flight day 150), supporting early in-flight retinal thickness measurements as a practical risk-stratification tool [89].

While asymmetric choroidal expansion typically resolves within 30 days of return to Earth, asymmetric optic disc morphological alterations and globe flattening have been documented to persist for at least 630 and 660 days, respectively [27], suggesting that anteriorly directed forces transmitted along the optic nerve sheath can induce durable scleral remodeling [90]. The slow resolution of structural changes post-mission may be attributed to the inefficiency of the posterior glymphatic system, which clears fluid at a much slower rate than anterior ocular drainage pathways, prolonging tissue thickening after gravitational gradients are restored [91].

Overall, approximately 70% of crew members involved in long-duration space missions have demonstrated at least one indicator of Spaceflight Associated Neuro-ocular Syndrome (SANS) [84]. SANS is therefore best characterised as a delayed-onset, cumulative pathology of LDSF, with symptoms typically emerging in mid- to late-mission phases and potentially conferring long-term consequences for ocular health and astronaut performance on interplanetary missions. Notably, to date, there have been no documented cases of uncorrectable visual acuity loss or permanent alterations in visual fields during or following long-duration spaceflight [84].

### 6.2. Pathophysiology of SANS

SANS arises from a convergence of altered pressure gradients, ocular biomechanics, venous congestion, and impaired glymphatic clearance under prolonged microgravity—not from ICP elevation alone. Cephalad fluid shift and venous congestion alter the TLPG and disrupt CSF compartmentalization within the optic nerve sheath, impairing glymphatic outflow and causing perivascular fluid accumulation. The resulting mechanical and hemodynamic stress drives neuroimmune activation, microstructural remodeling, and cellular injury in the choroid, ONH, and retina.

#### 6.2.1. Mechanical Forces and Pressure Gradients

CSF becomes compartmentalized within the orbital optic nerve sheath, producing localized pressure elevations independent of global ICP [5,92,93]. Imaging suggests mechanical traction: upward brain and optic chiasm displacement during microgravity pulls the optic nerve posteriorly, compressing the globe and altering the TLPG [17]. Finite element modeling predicts that nearly half of the astronaut population may experience extreme retrolaminar optic nerve strains due to a Poisson effect, where radial compression from elevated ICP induces a secondary longitudinal stretch [94]. Notably, upward displacement of the optic chiasm (0.39 ± 0.50 mm) among 22 astronauts of the ISS suggests brain movement unrelated to SANS development [77]. Interindividual variability in tissue stiffness, specifically a soft pia mater or optic nerve, is identified as a primary risk factor for the extreme deformations that likely trigger SANS-related tissue remodeling [94]. The intense shear stress generated by the TLPG can trigger phenotypic adaptation: venous endothelial cells in the posterior lamina cribrosa transform into a spindle-shaped appearance characteristic of arterial endothelium [95].

#### 6.2.2. CSF Dynamics and Glymphatic Failure

Glymphatic dysfunction with impaired perivascular CSF outflow is thought to promote fluid stasis within the prelaminar ONH, contributing to optic disc swelling [8,17,24,91,96]. A proposed dual mechanism underlies this: microgravity-induced pressure difference, impairing the efflux of ocular metabolites and fluids into the retrobulbar compartment and causing fluid to accumulate at the ONH [91].

The “bottleneck” hypothesis proposes that the optic canal acts as a site of localized compartment syndrome, restricting CSF exchange between the intracranial and orbital subarachnoid spaces, sharing clinical similarities with the impaired orbital venous return and facial edema seen in terrestrial prone-position spine surgeries [25,97]. Localized optic disc edema may also result from the mechanical forcing of retrobulbar CSF into the nerve parenchyma along the perivascular spaces surrounding the central retinal vessels. This infiltration may be facilitated by hydrostatically driven flux through microscopic pores in the arachnoid and pia mater, potentially aggravated by meningothelial cells proliferation in response to chronic orbital pressure [32].

#### 6.2.3. Vascular Mechanisms

Venous congestion with elevated vortex vein pressure drives choroidal expansion and folds [18,19,98], while elevated venous sinus pressure also contributes [99]. Dysregulation of choroidal blood flow and increased venous pressure are proposed as primary drivers of the choroidal engorgement and subsequent structural folds [100]. Class activation map analyses further suggest that the RNFL, retinal pigment epithelium (RPE), peripapillary choroid, and anterior lamina structures play a central role in SANS pathophysiology [101].

Longitudinal in-flight data from 12 astronauts indicate that macular luminal volume increase by 25% from pre- to post- flight, suggesting that choroidal vessels expand to occupy a greater relative space within the tissue [102]. Stasis of CSF, lymphatic, and vascular flow may reduce local tissue perfusion, leading to choroidal vasodilation and subsequent compression of the retinal pigment epithelium capillaries [103].

Reduced microvascular patterning was observed in 11 of 16 astronauts retinas. This may be driven by systemic spaceflight hypovolemia or a localized compartment syndrome, where excessive edematous fluid pressure overwhelms the endothelium’s ability to maintain fluid balance, potentially leading to vessel dropout [104].

Elevated CO_2_ levels on the ISS (typically 3–10 times higher than on Earth) aggravate these vascular contributions by increasing cerebral blood volume pulsatility and compromise venous drainage [5,17]. Elevated CO_2_ has also been associated with both headaches and greater SANS severity [17,99,105].

#### 6.2.4. Cellular and Molecular Changes

At the cellular level, reduced Na^+^/K^+^-ATPase activity secondary to oxidative stress and inflammation associated with venous stasis may contribute to edema by conditioning the tissue for transcapillary fluid movement, however this hypothesis is based on a low level of evidence [19,106].

A comparative analysis of 28 astronauts serum indicates that astronauts experiencing SANS symptoms exhibit a unique immunological profile characterised by higher levels of pro-inflammatory IL-6 and lower levels of anti-inflammatory IL-10 than those who remain unaffected [70,107]. Murine models further suggests that the transition back to a gravitational environment triggers significant cellular stress, as evidenced by a transient increase in retinal caspase-3 and 8-OHdG levels [70].

#### 6.2.5. Evidence from Animal Models

Animal models of spaceflight (murine tail suspension, rabbit head-down tilt, rat blast injury, dry immersion) cannot replicate true microgravity, as their anatomy, time scales, and stressors differ from those experienced by astronauts. Nonetheless, they offer mechanistic insight that orbital studies cannot provide.

In murine simulated weightlessness, the TLPG shifts from negative to positive, correlating with reduced ERG b-wave and photopic negative response amplitudes. The evidence points to a cascade: microgravity-induced mechanical stress activates central neuroimmune pathways, astrocytes shift phenotype, microglia respond, the outer nuclear layer thins, and retinal ganglion cells are lost [108]. The same model also produces persistent, asymmetric RNFL thinning [37].

Rat blast injury studies show that under normal gravity, autophagy and ER-phagy protect the retina after explosive trauma. In simulated microgravity, those repair pathways break down, raising the risk of irreversible retinal lesions [109]. Long-term simulations indicate that sustained cephalad fluid shifts lead to progressive optic nerve demyelination, loss of mature oligodendrocytes, microglial inflammation, and drive time-dependent retinal ganglion cell loss [110].

Overall, cephalad fluid shifts in microgravity remain the dominant proposed driver of SANS. Jugular venous distension, orbital venous congestion, and increased vortex vein pressure are implicated in optic disc edema and posterior globe flattening [19,22,33]. Elevated ambient CO_2_ exacerbates cerebral vasodilation, venous stasis, and vascular permeability [5,17,24].

### 6.3. Differentiation of SANS from Idiopathic Intracranial Hypertension

Although SANS shares optic disc edema as a feature with idiopathic intracranial hypertension (IIH), the two conditions are clinically and biomechanically distinct. Astronauts with SANS rarely experience headaches, pulsatile tinnitus, diplopia, or transient visual obscuration, which are typical of terrestrial IIH [19,87]. In SANS, ocular deformations are smaller in magnitude, frequently bidirectional, and biomechanically distinct from the large anterior displacements seen in IIH [8]. Retinal nerve fibre layer thickening in SANS is modest (~108 µm) compared with IIH (~300 µm), and Bruch’s membrane deformations are bidirectional rather than anterior [8,83]. Unlike IIH, long-duration spaceflight induces a unique posterior deepening of the Bruch membrane opening height that may persist as a residual recession in veteran astronauts, even before they embark on subsequent missions [111]. Consistent with this, baseline nasal retinal thickening has been observed in veteran crew members compared with non-flying controls, suggesting incomplete structural recovery after return to Earth [111]. Choroidal folds are most common in ISS crew members (24% of eyes with early ODE), whereas they are the least common fold type in IIH patients (10%) [85].

Longitudinal data from 19 crew members demonstrate that while mission-duration-adjusted retinal thickening shows a weak association with lateral ventricle expansion, the development of optic disc edema is largely uncoupled from changes in the broader intracranial compartment [112]. Comparisons with idiopathic intracranial hypertension (IIH) suggest that, unlike IIH, SANS pathophysiology likely involves compartmentalised cerebrospinal fluid pressures and orbital glymphatic dysfunction rather than uniformly elevated ICP, helping explain its asymmetry and persistence post-flight [5,8,85].

In conclusion, given this distinct profile SANS should not be considered a variant of classic IIH as shown in Table 3. Interventions that lower ICP may not address the compartmentalized CSF dynamics and orbital glymphatic dysfunction that are thought to drive optic disc edema in SANS and could even worsen the TLPG if IOP is simultaneously elevated. Countermeasure development should instead target the unique biomechanical and fluid-shift drivers of SANS.

### 6.4. SANS Risk Modifiers and Susceptibility

Systemic, genetic, anatomical, metabolic, and environmental factors modulate individual susceptibility to SANS, explaining the marked inter-astronaut variability observed in clinical outcomes.

Systemic modifiers under investigation include anaemia, altered ocular glymphatic clearance, high-salt diets, and CO_2_ exposure, all of which may influence translaminar pressure dynamics and ONH biomechanics [17,20,113]. Demographic and physical factors, such as age, sex, BMI, body weight, and prior spaceflight experience, appear less predictive [85,114].

Genetic variation in one-carbon metabolism pathways appears to modulate susceptibility to Spaceflight Associated Neuro-ocular Syndrome, based primarily on small head-down tilt bed-rest cohorts: individuals carrying a greater number of risk alleles such as MTRR 66G and SHMT11420C demonstrate significantly increased RNFL thickness and a higher incidence of optic disc oedema following head-down tilt bed rest in a 22 subjects cohort [115,116], especially when combined with low circulating levels of B vitamins. In contrast, the protective SHMT1 1420 TT genotype appears to mitigate risk in a small 11 subject head down tilt bed rest series [4,19]. Severe cases remain rare but instructive: a female astronaut of ISS mission with an exceptional combination of both genetic susceptibility (MTRR 66 and SHMT1 1420 alleles) and anatomical predisposition of preexisting pigment epithelium detachment (PED) exhibited the largest reported peripapillary retinal thickening and hyperopic shift to date, with partial in-flight improvement possibly related to B-vitamin supplementation or reduced cabin CO_2_ [117].

Anatomical features, including smaller optic nerve cup size including smaller cup volume, shallower cup depth and narrower cup width and choroidal thickness at the upper level of normal, may predispose to greater increases in peripapillary total retinal thickness (TRT), a sensitive objective measure for detecting ODE [114,117,118], thus a preflight optic cup volume of 0.3 mm^3^ or greater might be protective against ODE development [114]. Other anatomical features, such as overall preflight total retinal thickness, minimum rim width, Bruch membrane opening (BMO) area, RNFL thickness, choroid thickness, axial length, or refractive error, were not associated with ODE development [114]. The anatomical rigidity of the optic nerve sheath and the diameter of the optic canal may serve as critical risk modifiers, with high sheath stiffness and wider canal dimensions potentially facilitating greater pressure transmission to the posterior globe [91].

Repeat-mission data indicate that ocular responses are cumulative: a cohort study of 16 astronauts who completed two LDSFs from 2007 to 2024 shows that the magnitude of optic disc edema during an astronaut’s initial flight—specifically the change in peripapillary total retinal thickness within 250 µm of the Bruch membrane opening—strongly predicted the degree of retinal thickening in the same eye during subsequent missions [119]. A case report further demonstrates progression from unilateral choroidal folds and cotton-wool spots to bilateral folds and new-onset optic disc edema across successive flights in a 57-year-old astronaut [120].

### 6.5. In-Flight Monitoring and Diagnostic Toolkit

Imaging has been central to recognising and characterising SANS. The toolkit falls into three tiers: flight-ready core diagnostics, advanced imaging still being validated in space, and AI/computational tools that reduce reliance on subjective analysis.

#### 6.5.1. Core Operational Tools (Flight-Ready)

Optical coherence tomography (OCT) is the primary ISS diagnostic, capable of quantifying retinal nerve fibre layer thickness, posterior Bruch’s membrane opening (BMO) displacement, and choroidal folds with high precision [8]. An increase in total retinal thickness (TRT) greater than 19.4 µm, measured from the BMO to a 250 µm retinal eccentricity, is the threshold for early optic disc edema. Fundoscopy remains widely used but it is limited: evaluation of optic disc edema (ODE) by the Frisén scale is subjective, with up to 22% disagreement between graders, making it poorly suited for detecting the subtle, predominantly grade 1 ODE that characterises SANS [9,121]. Lastly, in-flight orbital ultrasonography provides real-time in-flight optic nerve sheath diameter (ONSD), globe flattening, and tortuosity assessment, with lower than terrestrial MRI and higher operator dependence [22,122].

#### 6.5.2. Advanced Imaging and Emerging Modalities

Advanced capabilities, including enhanced depth imaging OCT and OCT angiography (OCTA), have expanded in-flight assessments. OCTA was introduced onboard in 2018, holding promise for understanding vascular contributions to SANS [123]. Additionally, Lomb-Scargle periodograms applied to OCT videos detect heart-rate-dependent choroidal fluctuations, enabling precise vascular adaptation monitoring [102].

Post-flight high-resolution MRI enables measurement of ONSD, a widely used marker of intracranial pressure and automated segmentation reveals that following LDSFs the ONH shifts anteriorly by approximately 200 μm [124]. Moreover, it can document posterior globe flattening, pituitary concavity, and empty sella changes apart [3,122]. Its limitations include being costly and non-portable [122].

Many emerging modalities remain in the research phase, not yet validated for spaceflight, with sensitivity, specificity, and operational practicality unestablished. Among others, near-infrared spectroscopy (NIRS) enables non-invasive longitudinal glymphatic monitoring via CSF water content and intracranial pulsatility [123] and diffusion tensor imaging (DTI) probes cerebral and ocular microstructural integrity [125]. Beyond ocular imaging, non-invasive ICP monitoring through tympanic membrane displacement has been tested in microgravity analogues [41]. A comprehensive evaluation of emerging AI-driven frameworks, deep learning models, and computational mapping technologies used to enhance SANS detection is provided in Appendix B.

### 6.6. SANS Countermeasures (Mechanical, Environmental, Nutritional)

Given the clinical significance of SANS for astronaut vision and mission performance, multiple countermeasures have been proposed, although none are yet fully validated. The prevailing hypothesis suggests that mechanical countermeasures capable of transiently reversing the chronic headward fluid shift may be the most effective approach to prevent or mitigate SANS [84].

The most established countermeasure is the use of “space anticipation glasses”, plus lenses issued pre-flight, to provide immediate functional correction for the hyperopic refractive shifts that occur in-flight [41,97]. Beyond optical correction, in-flight reduction of ambient CO_2_ levels, noted to improve peripapillary thickening in at least one astronaut, suggesting that existing environmental control systems already contribute to SANS mitigation [117]. These measures are operationally routine and carry minimal additional mass or crew-time cost.

The most widely investigated mechanical countermeasure is lower body negative pressure (LBNP), which applies subatmospheric pressure (typically 20–30 mmHg) around the lower extremities to redistribute fluid toward the lower body, thereby mimicking the effects of gravity [103,126]. In head-down tilt analogs, LBNP has been shown to reduce optic nerve sheath distension and optic disc thickening [4,118], and simulation studies suggest that nightly (8-h-long) administration of low-level LBNP may influence choroidal morphology and Bruch’s membrane opening-minimum rim width [127]. However, acute in-flight application of 25 mm Hg for 10 to 20 min was insufficient to reverse structural ONH deformations, suggesting that longer-duration exposure may be needed [75]. Moreover, a 30-day head-down tilt bedrest study comparing cephalad fluid shift countermeasures found that LBNP alone did not prevent optic disc edema (ΔTRT250 > 20 μm in 11 LBNP subjects), whereas intermittent upright posture, which provides a complete reversal of the headward fluid shift, maintained ΔTRT250 below the NASA threshold for edema [118].

Artificial gravity protocols represent another analog-tested avenue. Data from JAXA’s Multiple Artificial-Gravity Research System (MARS) demonstrate that 1 G centrifugal loading can partially mitigate the 64% increase in retinal vascular endothelial cell apoptosis observed in microgravity, while also normalizing protein expressions linked to metabolic stress [83,128,129]. Identifying a specific ‘G-threshold’ for ocular protection could enable artificial gravity as a synergistic tool [129].

Other approaches target pressure gradients and venous return [103,130]. Interventions designed to modify TLPG have also been tested in short-duration analog settings. Pressurized or swim goggle can transiently raise IOP to counterbalance increased optic nerve sheath pressure and preserve lamina cribrosa biomechanics, as shown in 15°, 15-min head-down tilt experiments [4,40]. Impedance threshold devices, which reduce intrathoracic pressure and enhance venous return, have also been proposed as a means of modulating central venous and intracranial pressures, although their role in SANS remains largely experimental [21,130,131].

Several approaches remain at the conceptual or preclinical stage. Specialized multi-pressure dial goggles are being developed to apply precise negative or positive pressure to the orbital region using a calibrated pump but have not yet been studied in any analog or spaceflight setting [132]. Photobiomodulation using near-infrared or red light targets mitochondrial chromophores such as cytochrome c oxidase to stimulate ATP production and cellular respiration, potentially addressing the high energy demands of retinal ganglion cell axons at the ONH but has not been tested in microgravity [133].

On the nutritional and genetic side, B-vitamin supplementation (riboflavin, pyridoxine, folate, methylcobalamin) may correct one-carbon pathway deficiencies linked to risk alleles (MTRR 66G, SHMT1 1420C) and reduce susceptibility to optic disc edema [19,115,117]. Pharmacological interventions for elevated intracranial pressure, such as acetazolamide, must be used with caution in the space environment due to the increased risk of renal calculi formation stemming from drug-induced dehydration [41]. Artificial gravity regimens beyond the short MARS experiment, including optimal G-load, duration, and frequency for ocular protection, remain undefined and require further study [5,19].

Although no single countermeasure has demonstrated complete protection, the combination that currently appears most promising, despite its limited evidence, is nightly LBNP to reverse cephalad fluid shifts, paired with strict in-flight CO_2_ control and B-vitamin optimization in genetically at-risk individuals, with artificial gravity as a synergistic backup for exploration-class missions. A multi-modal approach integrating mechanical, nutritional, and environmental interventions will likely be necessary, and future missions to Mars will require dedicated mitigation strategies given SANS classification as a NASA mapped risk [84]. Operational guidelines, including a proposed ‘minimum kit’ for in-flight monitoring and specific trigger-based thresholds for deploying these countermeasures, are detailed in Appendix C.

## 7. Terrestrial Analogues (Head-Down Tilt Bed Rest, Dry Immersion, Limb Suspension, Parabolic Flight)

Terrestrial microgravity analogues, such as head-down tilt bed rest, dry immersion, or limb suspension reproduce cephalad fluid shifts but not radiation or full mission complexity [134].

Head-down tilt bed rest (HDTBR) typically places healthy volunteers in a 6° head-down position for days to weeks to simulate the cephalad fluid shift of microgravity and thereby reproduce many cardiovascular and neuro-ocular changes seen in orbit. In 6° HDTBR, approximately 45% of subjects developed Frisén grade 1–2 optic disc edema over 30 days [92,135] and peripapillary retinal thickness increased by about +11.5 µm over 70 days, often with reduced contrast sensitivity [30,136,137].

6° HDT, in the absence of elevated ambient CO_2_, produces a subtle optic disc edema, dilation of the ONSD, chorioretinal folds, and increased retinal thickness in otherwise healthy volunteers [136,138], and in strictly controlled HDT-only conditions a mean increase in total retinal thickness of 35.9 µm was observed on day 58, with 6 participants developing choroidal folds, retinal folds, and/or peripapillary wrinkles [136]. Even 3 h of head-down tilt measurably enlarges ONSD [139]. Nevertheless, exposure duration clearly modulates the magnitude of these responses, as longer-duration HDTBR (70 days) produces greater peripapillary retinal thickening than shorter exposure (14 days) [135].

30 days of strict HDTBR, compared with spaceflight of similar duration, induces greater optic disc edema (mean difference 37 µm) but less choroidal thickening (mean difference 27 µm) [88,92]. These results indicate differential vascular compliance between analogue and flight conditions. Despite these limits, HDTBR remains the most relevant terrestrial model for studying SANS mechanisms, risk factors, and countermeasures.

Another terrestrial microgravity analogue, dry immersion, also reproduces key aspects of the microgravity-induced cephalad fluid shift by immersing subjects in thermoneutral water while physically separated from direct water contact by a waterproof fabric, thereby unloading the body and reducing support from below. In one study, five days of dry immersion in women caused slight reductions in ocular axial length (from 23.29 to 23.21 mm), thickening of the peripapillary retinal nerve fibre layer (from a mean of 103.0 to 103.7 µm) and macular retinal thickness (from 265.9 to 266.1 µm)—alterations consistent with early stages of spaceflight associated neuro-ocular syndrome (SANS) but causing only modest visual alterations [140]. These findings support the use of dry immersion as a complementary analogue to HDTBR, particularly for probing early, subtle neuro-ocular and structural changes under conditions of more complete body unloading.

Lastly, parabolic flight serves as an important terrestrial analogue to study acute physiological changes induced by microgravity without requiring actual space travel [141]. This model provides repeated, transient exposure (up to ~20–40 s per parabola) to microgravity interspersed with alternating hypergravity phases, enabling investigation of immediate cardiovascular, vestibular, and ophthalmic responses, including changes in intraocular pressure, ocular geometry, and cerebrovascular dynamics [141].

Analogs presented in Table 4 provide the critical intermediate step between bench research and flight validation. LBNP, for example, was refined through HDTBR studies that demonstrated its ability to reduce optic nerve sheath distension and optic disc thickening before being tested aboard the ISS [127]. The shorter and more controlled exposure of dry immersion makes it suitable for early-stage screening of fluid-shift countermeasures, while parabolic flight allows rapid iteration of acute interventions such as pressurized goggles or impedance threshold devices. No single analog captures the full spaceflight environment, but their combined use—longer HDTBR for structural endpoints, dry immersion for unloading-specific effects, parabolic flight for acute physiological responses—provides a rational, staged pathway for maturing SANS countermeasures before operational deployment.

## 8. Long-Term Ocular Risks

### Cataractogenesis and Radiation-Associated Risk

Cataractogenesis is a recognised late effect of space radiation, influenced by radiation quality, age, sex, hormonal milieu, and genetic background [142,143,144]. Ionizing radiation is a potent cataractogen because the lens, being avascular and relatively hypoxic, has limited capacity to repair radiation-induced molecular damage. High-LET (linear energy transfer) heavy ions, such as iron ions and other HZE (high—H, atomic number—Z, and energy—E) particles in galactic cosmic rays deposit energy densely along their tracks, generating clustered DNA lesions, modifying gene expression and protein damage in lens epithelial cells, which overwhelms antioxidant defenses and DNA repair pathways [11,12], increases oxidative protein modifications and prematurely triggers fibre cell differentiation [145,146]. In contrast, low-LET protons, which are far more abundant in galactic cosmic rays, are thought to injure the lens mainly through the oxygen effect and chronic oxidative stress [15]. Molecular studies support these clinical findings: heavy ion radiation alters gene and protein expression in lens epithelial cells [145].

In the Longitudinal Study of Astronaut Health (LSAH) by NASA, US astronauts whose cumulative space lens dose exceeded 8 mSv (mean 45 mSv) showed a significant hazard ratio for “all” cataracts at age 60 or 65 compared with those whose dose stayed below 8 mSv (mean 3.6 mSv) [21,71]. In the NASA longitudinal cataract study, astronauts with mission-related lens doses of 15–129 mSv had a 2.2-fold higher risk of posterior subcapsular cataracts (PSC) and more extensive cortical opacities than non-flyers [6]. The NASA Study of Cataract in Astronauts (NASCA) confirmed that cortical opacification progresses more rapidly with increasing cumulative space radiation exposure, with a median progression rate of about 0.25% of lens area per Sv per year [6,7]. By contrast, PSC changes showed only a borderline association with dose (*p* = 0.056), and nuclear cataracts were more strongly linked to age than to radiation exposure [7]. Animal studies support these findings: exposure to ^56^Fe accelerates cataract onset compared with low-LET X-rays in rats, and ageing amplifies this effect—one-year-old animals develop lens opacities faster than younger cohorts [12,13].

Radiation measurements during Artemis I provide additional context for risk modelling. In the Orion spacecraft, shielded regions recorded proton-belt dose rates as low as 69 μGy/min, while the least protected zones reached 287 μGy/min, highlighting the importance of spacecraft design for exposure reduction [147]. Even though interplanetary galactic cosmic ray dose equivalents in Orion were up to 60% lower than earlier estimates, a Mars mission is still expected to exceed NASA’s current career dose limit of 600 mSv [148].

Cataract type may depend not only on radiation quality but also on ambient oxygen levels [6,7]. Earlier spacecraft atmospheres with 70–100% oxygen likely increased oxidative stress in the lens and may have contributed to nuclear cataract risk. In contrast, modern capsule designs maintain lower oxygen tensions, closer to the physiological “Goldilocks range” for lens epithelial cells [15], which may mitigate some of this oxidative burden.

Genetic predisposition in murine models further modifies susceptibility: Rad9^+^/^−^ and Atm^+^/^−^ mice develop cataracts earlier than wild-type animals even without irradiation, and cataract progression is faster after exposure [16]. Male rodents appear more vulnerable than females [143], although this sex effect has not yet been demonstrated in astronauts. The role of oestrogen is also complex: supplementation protects against cataracts induced by low-LET photons [13,14] but offers little benefit and may even accelerate changes after heavy-ion exposure [14]. These divergent effects may reflect differences in DNA damage quality and in lens oxygen gradients that modulate oxidative stress [15].

With cataracts now recognized alongside cancer and degenerative disease as a major long-term radiation risk, mitigation strategies are a priority. Potential countermeasures fall into three categories: physical shielding, pharmacological agents and clinical management. Physical shielding, such as storm shelters within Orion, can limit acute solar particle event exposures to <150 mSv [147], but is impractical against chronic galactic cosmic rays. Pharmacological approaches, including antioxidants (vitamin E, selenium, melatonin), radioprotectors such as amifostine, and PrC-210, are under investigation, however, no pharmacological agent is currently approved or routinely used for cataract prevention in astronauts [144,149,150]. Clinical management focuses on early detection and optimized treatment pathways. Imaging technologies such as ultrasound biomicroscopy (UBM) offer non-invasive, in-flight monitoring of early lens changes, supporting timely decision-making [10]. Cataract surgery with intraocular lens (IOL) implantation is compatible with subsequent spaceflight: astronauts with IOLs have completed both short- and long-duration missions without visual compromise, although the presence of an IOL currently remains disqualifying for astronaut selection [151].

Although cataract prevalence remains low within the relatively young astronaut cohort, cumulative radiation exposure in exploration-class missions will likely accelerate cortical and PSC lens opacification. Cataractogenesis, therefore, represents not only a predictable late effect of deep-space radiation but also a significant barrier to truly autonomous medical care during such missions. This underscores the need for international longitudinal surveillance, studies at realistic low dose rates, and the integration of real-time ocular monitoring into mission medical protocols.

## 9. Limitations

The spaceflight ophthalmology evidence base remains constrained by small sample sizes, heterogeneity of missions and protocols, and limited opportunities for controlled experimentation. Terrestrial analogues provide essential support, but do not fully reproduce the duration and complexity of exploration-class missions. Accordingly, conclusions should be interpreted as a synthesis across constrained datasets rather than definitive causal proof for single mechanisms.

## 10. Conclusions

Ocular health in space is a complex, multifactorial challenge that intersects with mission safety, crew autonomy, and long-term astronaut well-being. This review reveals that spaceflight induces a spectrum of ocular changes, from tear film instability and corneal stress [4,6,22,136], radiation-induced cataracts to posterior globe deformation and SANS [20,44,53]. SANS is a delayed, cumulative risk that requires early, objective monitoring. These effects are driven by a convergence of microgravity-induced fluid shifts [75], elevated ambient CO_2_ [46,56], radiation exposure [12,13,46,143,146], and individual susceptibility shaped by genetic and anatomical factors [115,116].

While current countermeasures, ranging from lower body negative pressure [127], nutritional supplementation [115], and imaging-based diagnostics [10,78,111,124,138,152] to AI-driven tools [101], offer promising avenues, no single intervention has yet proven fully protective. Integrated mechanical, environmental, and nutritional countermeasures are more realistic than single-level solutions.

As humanity prepares for interplanetary travel, preserving ocular integrity will be essential not only for individual astronaut health but also for the success of crewed missions beyond low Earth orbit. Continued research, longitudinal surveillance, and innovation in diagnostics and therapeutics will be key to meeting this challenge.

## Figures and Tables

**Table 1:** Mechanisms of SADES shared with terrestrial dry eye disease space-specific.

SADES Mechanisms:	
Shared Terrestrial and Spaceflight:	Spaceflight-Specific:
Reduced blinking frequency	Strong cabin airflow
Meibomian gland dysfunction	Elevated ambient CO_2_
Immune dysregulation/inflammation	Radiation exposure (ionising, HZE, SPE)
Tear film instability/evaporative loss	Microgravity-induced periorbital edema
Foreign body exposure	Orbicaularis oculi weakening
	Altered eyelid position (reverse ptosis, brow elevation)
	Ocular decompression sickness
	Lunar/Martian dust toxicity
	Altered tear dynamics

**Table 2:** Structural ocular changes associated with LDSF and their potential functional consequences.

Structural Change	Possible Functional Consequence
Axial length reduction (0.05–0.08 mm)	Hyperopic shift
Anterior lens displacement	
Anterior chamber volume reduction	
Peripapillary choroidal thickening	Possible contributor to hyperopic shift
ONH/disc swelling	Impaired fine visual acuity
Globe flattening	Reduced dynamic visual acuity during G-transitions [82]
Peripapillary choroidal thickening and ONH edema	Possible visual field deficits
Optic nerve elongation (0.8 mm mean)	May affect ONH compliance; correlates with SANS severity

**Table 3:** Comparative Features of Spaceflight Associated Neuro-Ocular Syndrome (SANS) and Idiopathic Intracranial Hypertension (IIH).

Feature	SANS	IIH
Symptoms	Rare headaches, tinnitus, diplopia, or visual obscurations	Frequent headaches, pulsatile tinnitus, diplopia, transient visual obscurations
ICP profile	Normal or mildly elevated; compartmentalised CSF pressures suggested	Uniformly elevated ICP (typically >25 cm H_2_O)
RNFL thickening	~108 µm (modest)	~300 µm (marked)
Bruch’s membrane opening deformation	Bidirectional (some posterior deepening); residual recession in veterans	Large anterior displacement
Choroidal folds	Common (24% of eyes with early ODE)	Least common fold type (10%)
Symmetry	Often asymmetric; can be unilateral	Typically bilateral and symmetric
Recovery after Earth return	Variable; incomplete structural recovery documented	Usually resolves with ICP lowering

**Table 4:** Comparative summary of terrestrial microgravity analogues.

	HDT Bed Rest	Dry Immersion	Parabolic Flight
Typical exposure duration	Days to weeks (3 h to 70 d in studies)	Days (5 d in the cited study)	Seconds per parabola (20–40 s), repeated
Main physiological features reproduced	- Cephalad fluid shift (6° HDT)	- Complete body unloading - Fluid shift - Reduced axial support	- Transient microgravity (alternating with hypergravity) - Acute fluid shifts
Key ocular outcomes	- Optic disc edema (Frisen 1–2, ~45% at 30 d) - Peripapillary TRT increase (~35.9 um at 58 d) - ONSD dilation - Chorioretinal folds - Reduced contrast sensitivity	- Slight axial length reduction (23.29 to 23.21 mm) - RNFL thickening (103.0 to 103.7 um) - Macular thickening (265.9 to 266.1 um)	- Acute IOP changes - Transient ocular geometry shifts - Cerebrovascular dynamics
Strength	- Long-duration exposure (up to 70 d) - Reproduces many SANS features (ODE, ONSD dilation, chorioretinal folds, retinal thickening) - Standardised protocol - Reliable cephalad fluid shift - Well-suited for countermeasure testing	- More complete body unloading than HDTBR (no axial support) - Good for probing early subtle changes - Shorter study duration - Complements HDTBR	- Captures immediate/acute responses - No confinement burden - Allows rapid iteration within single session - Repeatable across many parabolas
Limitations	- Greater ODE than flight (mean +37 um) but less choroidal thickening (mean −27 um) - Imperfect tissue compliance match - Subject confinement burden	- Modest ocular changes - Limited validation data	- Exposure too brief for any structural remodelling - Alternating hypergravity confounds chronic adaptation - Motion sickness in some subjects

**Figure A1:**
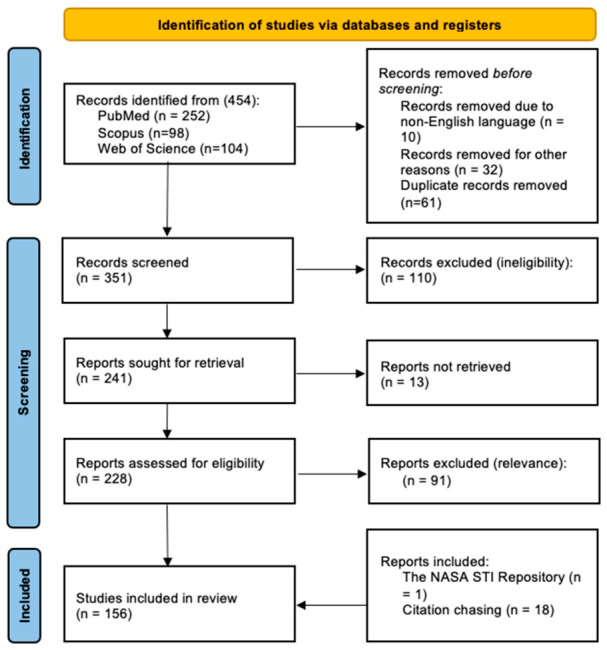
PRISMA 2020 flow diagram illustrating the identification, screening, and inclusion of studies across databases and supplementary sources for the systematic review.

## Data Availability

Data generated or analyzed during the feasibility measurement are included in this published article and its appendices.

## Appendix A

### Appendix A.1. Search Strategy and PRISMA Details

The primary aim of this study was to conduct a review of the scientific literature concerning ocular symptoms associated with long-duration spaceflight (LDSF). Overview of the research method, based on the PRISMA flow diagram. Summary of the different research steps followed to choose the final included studies. We reviewed literature using the PubMed, Scopus and Web of Science Core Collection database. Search terms included “spaceflight”, “astronaut”, “dry eye syndrome”, “ocular biometry”, “corneal edema”, “cataractogenesis”, “cataract” or “spaceflight associated neuro-ophthalmic syndrome” combined with terms: “astronaut” or “spaceflight”. Only English-language articles published from January 2006 to December 2025 were considered. Inclusion criteria included peer-reviewed original research, systematic reviews, meta-analyses, and NASA technical reports published between January 2006 and December 2025. Experimental analogue and animal studies were included when they provided mechanistic or physiological insight relevant to spaceflight associated ocular alterations. Due to the limited number of human spaceflight studies and the inherently restricted astronaut sample sizes available in the literature, no minimum sample size threshold was applied. Exclusion criteria included non-English publications, conference abstracts without accessible full text, editorials, duplicate records, and studies not directly related to ocular outcomes or spaceflight associated physiology.

### Appendix A.2. Full Search Strategies

PubMed/MEDLINE (template)

(“spaceflight”[tiab] OR astronaut*[tiab] OR micrograv*[tiab] OR “long-duration”[tiab] OR “head-down tilt”[tiab] OR “dry immersion”[tiab] OR “parabolic flight”[tiab]) AND (ocular[tiab] OR eye[tiab] OR ophthalm*[tiab] OR vision[tiab] OR “optic disc”[tiab] OR “optic nerve”[tiab] OR retina*[tiab] OR choroid*[tiab] OR SANS[tiab] OR “neuro-ocular”[tiab] OR cataract*[tiab] OR “dry eye”[tiab]) AND (“2006/01/01”[dp]: “3000”[dp]).

Scopus (template)

TITLE-ABS-KEY (spaceflight OR astronaut* OR micrograv* OR “long-duration” OR “head-down tilt” OR “dry immersion” OR “parabolic flight”) AND TITLE-ABS-KEY (ocular OR eye OR ophthalm* OR vision OR “optic disc” OR “optic nerve” OR retina* OR choroid* OR SANS OR “neuro-ocular” OR cataract* OR “dry eye”) AND PUBYEAR > 2005.

Web of Science Core Collection (template)

TS = (spaceflight OR astronaut* OR micrograv* OR “long-duration” OR “head-down tilt” OR “dry immersion” OR “parabolic flight”) AND TS = (ocular OR eye OR ophthalm* OR vision OR “optic disc” OR “optic nerve” OR retina* OR choroid* OR SANS OR “neuro-ocular” OR cataract* OR “dry eye”) Refined by: DOCUMENT TYPES = (ARTICLE OR REVIEW) AND LANGUAGES = (ENGLISH) AND PUBLICATION YEARS = (2006–2025).

## Appendix B. Computational Approaches

The subtlety of early SANS and in-flight acquisition constraints make standardized artificial intelligence supported analysis valuable for reliable trend-based decisions. However, many emerging technologies remain in the research phase, not yet validated for spaceflight, with sensitivity, specificity, and operational practicality unestablished.

Deep learning frameworks are already yielding results: SegFormer, a vision-transformer segmentation network retrained on astronaut OCT macular videos, identified a 25% post-flight increase in pulsatile luminal area fluctuations, possibly reflecting reduced ocular rigidity [102].

Generative adversarial networks (GANs) offer another practical step forward. FA4SANS-GAN synthesizes fluorescein angiography from standard fundus photographs, enabling detection of vascular leakage and maculopathy without intravenous dye [153], and Fréchet and Kernel Inception Distances confirm these GANs can be robust to acquisition noise and preserve the structural small retinal vessels and aneurysms better than generalized deep learning models [153].

Computational vascular analysis adds further resolution. Computational mapping using VESGEN (Vessel Generation Analysis) software (version 1.0) skeletonizes retinal vessels, assigns them to branching generations (Lv1–LvN), and quantifies generation-specific vessel density and patterning changes [104]. Its application has shown that spaceflight remodels retinal vessels in a way that is generation-specific: density decreases primarily in small vessels (Lv ≥5) while larger vessels remain preserved [104]. The Subclinical Vascular Pathology Index (SVPI) extends this logic by ranking microvascular loss in asymptomatic astronauts as a potential biomarker for future SANS susceptibility [104].

Non-invasive functional markers are also being developed. Governed by the TLPG, spontaneous venous pulsations (SVPs) offer a real-time non-invasive marker for TLPG assessment [154]. Virtual reality (VR) platforms now using Bayesian analysis detect visual changes more sensitively than traditional chart-based tests [123], and NASA supports a compact VR device combining vision assessments, computational mapping, and machine learning for in-flight monitoring [155]. The integration of spatial computing and 4K displays could enable astronauts hands-free diagnostics, reducing setup time, and improving precision of measurements, such as dynamic visual acuity [154]. XR platforms also show promise as non-invasive ICP surrogates via pupillary constriction metrics, though this remains research-stage [154].

The 30-pound ISS mass limit on in-flight medical hardware makes the development of compact multimodal platforms integrating these capabilities a priority [153]. AI-driven approaches to decision support may become integral to autonomous management of SANS in exploration-class missions [66,156].

## Appendix C. Implications for Spacecraft Design and Mission Operations

### Appendix C.1. Suggested Onboard “Minimum Kit” and Operational Footprint

A mission-relevant onboard capability should be defined by what must be measured (structure, function, and relevant physiologic proxies), balanced against mass, volume, power, and crew-time constraints. If only one or two devices are feasible, the minimal recommended configuration would consist of:

- OCT: A spectral-domain OCT (SD-OCT) with both posterior segment (ONH, RNFL, ganglion cell-inner plexiform layer, choroid) and anterior segment (corneal thickness, anterior chamber depth, lens) imaging capability.
- Ultrasound system: A portable unit with a high-frequency linear probe (7–15 MHz) for ONSD assessment and, where available, a low-frequency phased-array probe for cerebral and ocular hemodynamic evaluation.
- Routine functional testing (visual acuity, Amsler grid, contrast sensitivity) can be performed with existing crew medical equipment and software-based tools without additional stowage.

### Appendix C.2. Trigger-Based Operational Decision Support

Operationally, a trigger-based concept of operations is recommended in which objective trends prompt predefined responses:

- Total retinal thickness (ΔTRT) increase > 10%, Frisén grade ≥ 2 optic disc edema or ONSD > 15% increase from preflight baseline: Initiate enhanced monitoring (weekly OCT + ONSD ultrasound); optimize CO_2_ management; consider LBNP or thigh-cuff deployment sessions as a first mechanical countermeasure.
- Lens opacification progression: Flag for post-mission cataract surveillance; incorporate into individual long-term risk counseling.

Real-time thresholds should be refined through terrestrial analogue testing and validated against in-flight data as it accumulates.

### Appendix C.3. Design Levers, Evidence Gaps, and Verification Roadmap

Vehicle layout and shielding decisions influence radiation exposure, linking cataract risk to spacecraft/habitat design. Given persistent uncertainties and the constraints of the spaceflight evidence base, future work should prioritize harmonized endpoints, repeatable acquisition protocols, and integrated countermeasure trials. Terrestrial analogues (HDTBR, dry immersion, and parabolic flight) remain essential, but should be interpreted with careful attention to their limits in reproducing chronic exposure and mission complexity.

**Table A1:** Summary of ocular hazards, outcomes, detection methods, mitigation strategies, and verification approaches.

Hazard	Outcome	Primary Detection	Mitigation Levers	Verification Platforms
Fluid shift/venous congestion	SANS, refractive shift, mission impact	OCT trending + US/ONSD	LBNP, cuffs, posture, TLPG, CO_2_, nutrition	HDTBR, dry immersion, ISS
Cabin CO_2_ (elevated vs. Earth)	Modifier of posterior findings	Cabin telemetry + OCT/US	CO_2_ removal (design) + exposure management (ops)	HDTBR ± mild hypercapnia
Nutritional + genetic susceptibility (one-carbon pathway)	Stratified risk for ODE/SANS indicators	Preflight biomarkers/genotyping; enhanced monitoring	B-vitamin supplementation; tailored monitoring intensity	Cohort stratification: controlled trials needed
Airflow, Humidity, dust	Dry eye; infection; performance	Symptoms + tear metrics; functional testing	Environment optimization; lubrication	In-flight monitoring
Deep-space radiation (HZE/high-LET)	Cataractogenesis (late effect); programmatic burden	Lens surveillance + dosimetry	Shielding, storm shelter; exposure management	Astronaut cohorts, heavy-ion animal studies
Perfusion dynamics (OCTA/LSFG)	SANS trajectory marker	OCTA; LSFG (MBR)	Adjunct endpoint for response verification	HDT, parabolic analogues
